# Prevalence of Sports Injuries Among Athletes in the Lucknow Region: A Cross-Sectional Study

**DOI:** 10.7759/cureus.90266

**Published:** 2025-08-17

**Authors:** Abhishek Chowdhery, Abhishek Agarwal, Abhishek Saini

**Affiliations:** 1 Sports Medicine, King George's Medical University, Lucknow, IND

**Keywords:** athletes, contact sports, injury prevention, lower limb injuries, lucknow, prevalence, sports injuries, training safety

## Abstract

Sports-related injuries pose a critical concern, particularly among adolescents and young adults actively involved in athletic pursuits. In India, efforts to address sports-related injuries are hindered by the lack of comprehensive injury surveillance systems, underdeveloped sports medicine facilities, and inadequate awareness among athletes about effective injury prevention and management practices. This cross-sectional study aims to investigate the prevalence, distribution, and causes of sports injuries among athletes in the Lucknow region of Uttar Pradesh, India. Data were collected from 400 athletes aged 14-30 years enrolled across five prominent sports institutions, covering cricket, football, athletics, and wrestling. A combination of structured questionnaires and physical examinations was employed to gather injury-related information. The overall injury prevalence was 48.5%, with contact sports such as football (64%) and wrestling (58%) accounting for the highest rates. Lower limb injuries were predominant (61.3%), particularly knee sprains, ankle sprains, and hamstring strains. Gender-wise, male athletes experienced a slightly higher injury rate (50.6%) compared to female athletes (45.2%), though this was not statistically significant. Injuries were more frequently reported during training sessions than during competitions. The study found that inadequate warm-up routines, insufficient rest periods, and lack of access to sports medicine were key contributing factors. These findings underscore the pressing need for comprehensive injury prevention strategies, including structured warm-up protocols, regular sports medicine sessions, and education programs on injury management for both athletes and coaches. Establishing region-specific injury surveillance systems could help in developing effective, localized intervention programs. This study not only adds to the sparse epidemiological data on sports injuries in North India but also offers actionable insights for stakeholders to enhance athlete safety and long-term performance.

## Introduction

Sports and physical activities offer significant health benefits, including improved cardiovascular health, enhanced musculoskeletal function, and psychosocial well-being. However, they also carry the inherent risk of injury, particularly among competitive athletes and physically active youth [1]. Sports injuries constitute a major health burden, both globally and in India, affecting athletic performance and long-term physical health [2].

The incidence and severity of sports injuries are influenced by various factors such as sport type, intensity, frequency of participation, training quality, and availability of medical support [3]. Contact sports like football and wrestling inherently carry a higher risk of traumatic injuries, including ligament tears and dislocations, compared to non-contact disciplines such as athletics [4]. Inadequate training supervision, lack of protective gear, and overtraining are common risk factors, especially in developing nations where sports medicine infrastructure is still evolving [4].

Sports medicine is the need of the hour for comprehensive medical care of active-exercising individuals to weekend warriors and professional elite athletes [5]. An average annual estimate of 8.6 million sports- and recreation-related injury episodes was reported, with an age-adjusted rate of 34.1 per 1,000 population. Males (61.3%) and persons aged 5-24 years (64.9%) accounted for more than one-half of injury episodes [6].

In the Indian context, the sports landscape has undergone a significant transformation in recent years, with increased governmental and private investment in athlete development. Yet, this progress is not matched by advancements in injury surveillance, research, or prevention mechanisms [7]. It is indicated that more than 40% of youth athletes suffer at least one significant musculoskeletal injury per year, with knee and ankle injuries being the most prevalent. However, such data is mostly generalized for urban centers or national-level athletes, leaving a substantial gap in region-specific research, especially for cities like Lucknow.

Some recent Indian studies suggest that female athletes may be more prone to overuse injuries and joint instabilities, often due to anatomical and hormonal differences [8-11]. Lucknow, a prominent urban center in North India, hosts multiple sports academies and training centers. Despite the growing sports infrastructure and athlete population, empirical data on sports injury patterns in the region are lacking. This makes it challenging to implement informed preventive strategies, tailor injury rehabilitation programs, or advocate for improved medical facilities. Psychological factors also play a role in injury risk and recovery.

Athletes under pressure to perform may neglect rest and play through pain, increasing the likelihood of chronic injuries [10]. Epidemiological data are critical in identifying common injury types, high-risk sports, and susceptible demographic groups. Studies from other parts of India have shown that injury prevalence can range from 35% to 60%, with contact sports like football, hockey, and wrestling showing significantly higher rates [12]. Moreover, a significant proportion of these injuries occur during training due to poor warm-up practices, inadequate rest, or excessive workload.

Gender differences in injury patterns are also noteworthy. Although male athletes traditionally report higher injury rates due to aggressive play styles, rising female participation in competitive sports is changing this trend. Recognizing these patterns, the present study aims to fill the data gap for the Lucknow region by providing a detailed analysis of injury prevalence, distribution across sports and gender, and associated risk factors.

The specific objectives of this study are to estimate the prevalence of sports injuries among athletes in Lucknow, categorize injuries based on anatomical location and sport type, evaluate gender-based differences in injury patterns, identify training and recovery-related factors contributing to injuries, and propose actionable recommendations for injury prevention and athlete safety.

Findings from this study will offer critical insights for sports authorities, healthcare providers, and policymakers. Region-specific data can drive targeted interventions such as injury education programs, on-field sports medicine support, and improved training protocols. Moreover, this research can serve as a baseline for future longitudinal studies and help integrate sports injury monitoring into regional health planning.

## Materials and methods

Study design and setting

This study was designed as a cross-sectional observational investigation aimed at assessing the prevalence and patterns of sports-related injuries among young athletes. The study was carried out over a four-month period, from January to April 2025. It was conducted at five prominent sports academies and regional sports training centers located in Lucknow, Uttar Pradesh, India. These centers specialize in training athletes across a range of disciplines, including cricket, football, athletics, and wrestling. Each of these academies is recognized for its structured training programs and has a diverse population of athletes undergoing regular, intensive physical training. This cross-sectional observational study was conducted in accordance with the Strengthening the Reporting of Observational Studies in Epidemiology. For this study, a sports-related injury was defined as: “Any musculoskeletal or soft tissue damage sustained during sports participation, whether in training or competition, that resulted in (a) inability to participate fully in the next planned training session or competition (time-loss injury) or (b) required assessment/treatment by a healthcare professional (medical-attention injury).”

Selection criteria for the academic center

Selection criteria were as follows: (i) Offering structured, formal training programs in at least one of the following sports: cricket, football, athletics, or wrestling; (ii) a minimum enrollment of 50 active athletes aged 14-30 years; (iii) maintaining comprehensive injury and training records for at least the preceding 12 months; and (iv) on-site certified coaching staff and access to sports medicine professionals. Centers failing to meet these criteria were excluded.

Study population

Inclusion Criteria

Inclusion criteria were as follows: (i) Male or female, aged 14-30 years; (ii) actively engaged in formal sports training and competitive events at the time of the study; and (iii) ≥1 year of continuous participation in their primary sport before enrollment.

Exclusion Criteria

Exclusion criteria were: (i) A documented congenital musculoskeletal disorder; (ii) a chronic medical condition independently predisposing to injury (e.g., severe osteoarthritis, uncontrolled epilepsy); and (iii) incomplete or unverifiable injury/training record

Sample size and sampling technique

A total sample size of 400 athletes was determined to be adequate for the study. This was calculated based on an estimated injury prevalence of 50%, which provides the most conservative (maximum) sample size for a given confidence level. The confidence interval was set at 95%, and the margin of error was fixed at 5%. To ensure that all major sports disciplines were proportionally represented, a stratified random sampling technique was employed. Each sport category (cricket, football, athletics, and wrestling) served as a stratum, and participants were randomly selected within each group in accordance with their representation in the overall athlete population of the academies.

The sample size was calculated using the single proportion formula: this yielded n = 384; the sample was increased to 400 to account for a potential 4% non-response rate.

Data collection tools and procedure

Data were collected through a structured and validated questionnaire that was pre-tested for reliability and content validity. The questionnaire was administered in person by trained research assistants. It included multiple sections covering: (i) Structured Questionnaire which was developed from validated injury surveillance instruments, pre-tested in a pilot sample of 20 athletes; (ii) On-site Clinical Examination which was conducted by certified sports medicine professionals to verify self-reported injuries; (iii) Demographic Information: Age, gender, level of education, and duration of sports participation; (iv) Training Characteristics: Type of sport, weekly training hours, level of competition (district, state, national), and coaching intensity; (v) Injury History: Details on prior injuries, including anatomical location, type of injury (e.g., sprain, fracture, strain), mechanism of injury (traumatic vs. overuse), circumstances of occurrence (training vs. competition), and recurrence; and (vi) Treatment and Recovery: Type of medical attention received, duration of recovery, and return-to-play timelines.

Self-reported injuries were corroborated with on-site clinical examinations conducted by sports medicine professionals. Medical records were reviewed wherever available to enhance data accuracy.

A stratified random sampling approach was used. Each sport (cricket, football, athletics, wrestling) formed a stratum, and participants were randomly selected from each proportionate to their representation in the center’s athlete roster.

Informed consent

Prior to participation, all athletes were provided with a detailed explanation of the study's objectives, procedures, potential risks, and benefits. Written informed consent was obtained from each participant. For athletes under the age of 18, consent was obtained from a parent or legal guardian, along with assent from the athlete.

Statistical analysis

All collected data were coded and entered into IBM SPSS Statistics for Windows, Version 25 (Released 2017; IBM Corp., Armonk, New York, United States), for analysis. Descriptive statistics (means, standard deviations, frequencies, and percentages) were used to summarize demographic and injury-related variables. Inferential statistics were applied to examine associations between categorical variables using chi-square tests. The level of statistical significance was set at p < 0.05. Where appropriate, stratified analyses were conducted to identify sport-specific patterns or risk factors associated with injury prevalence. Missing demographic data <5% were addressed with complete-case analysis. Confounder adjustment and potential confounding variables were identified a priori based on previous literature and biological plausibility [13]. These included: (i) Demographic factors - Age (continuous), sex (male/female); (ii) Sport-specific factors - Type of sport (categorical), years of participation,(continuous), weekly training hours (continuous), coaching intensity (ordinal); and (iii) Competition level - District, state, or national level participation, a three-step approach: Bivariate screening, forced entry of key confounders and backward elimination for confounding variables.

## Results

A total of 400 participants were assessed, with an average age of 20.4 ± 3.2 years, indicating a relatively young population. The sample was predominantly male, comprising 60% of participants, while female participants made up 40%.

Overall, injuries were reported by 48.5% of participants, reflecting a significant incidence across the cohort. When analyzed by sport, football showed the highest injury prevalence (64%), mainly involving ankle sprains and anterior cruciate ligament (ACL) tears. Wrestling followed closely with a 58% injury rate, where shoulder dislocations were most frequent. In athletics, 42% of participants experienced injuries, predominantly hamstring strains. Cricket had the lowest injury rate at 30%, with common complaints including lower back pain and elbow tendinitis.

A total of 400 participants were included in the study, representing 100% of the sample population (Table 1). The mean age of the participants was 20.4 years with a standard deviation of 3.2 years, indicating a predominantly young adult cohort with relatively low variability in age.

**Table 1:** Demographic and Injury Prevalence Data

Demographics	Frequency (%)
Total participants	400 (100%)
Age (Mean ± SD)	20.4 ± 3.2 years
Male	240 (60%)
Female	160 (40%)
Overall injury prevalence	194 (48.5%)

In terms of gender distribution, 240 participants (60%) were male, while 160 participants (40%) were female, reflecting a male-dominant sample.

The overall prevalence of injuries among the participants was 48.5%, with 194 individuals reporting at least one injury during the study period. This suggests that nearly half of the study population experienced injuries, highlighting a substantial burden of injury in this group.

Injury prevalence varied considerably across different sports (Table 2). Football players demonstrated the highest injury rate, with 64% of participants reporting injuries. The most frequently observed injuries in this group included ankle sprains and ACL tears, both of which are common in high-impact and contact sports involving rapid directional changes. Wrestling had the second-highest injury prevalence at 58%. Shoulder dislocation was the most commonly reported injury in this group, consistent with the physical nature and intense upper-body engagement inherent to the sport.

**Table 2:** Injury Distribution by Sport and Type ACL: Anterior cruciate ligament

Sport	Injury Prevalence	Common Injury Types
Football	64%	Ankle sprain, ACL tear
Wrestling	58%	Shoulder dislocation
Athletics	42%	Hamstring strain
Cricket	30%	Lower back pain, elbow tendinitis

This study establishes that sports injuries are a significant health concern among young athletes in Lucknow, with an overall prevalence of 48.5%. The majority of injuries involved the lower limbs (61.3%), particularly ankle sprains, knee ligament injuries, and hamstring strains. Football and wrestling athletes experienced the highest injury rates (64% and 58%, respectively), reflecting the high physical contact, explosive movements, and repetitive joint loading intrinsic to these sports.

## Discussion

The findings from this study affirm that sports injuries represent a significant health burden among athletes in the Lucknow region, with a prevalence rate of 48.5%. This rate aligns closely with findings from North Indian cities such as Delhi and Chandigarh, where injury rates among adolescent and collegiate athletes range between 45% and 55% [7].

Lower limb injuries were most prevalent (61.3%), dominated by ankle sprains, knee ligament injuries, and hamstring strains. These findings are consistent with global literature, which identifies the lower extremities as particularly vulnerable in sports involving sprinting, jumping, and directional changes [3]. Sports like football and wrestling accounted for the highest injury rates, 64% and 58%, respectively, owing to their high physical contact intensity and repetitive joint stress.

Moreover, informal coaching practices without certified medical staff further compound injury risk. Certain training injuries in Indian athletes result from poor biomechanical techniques and a lack of preventive care. In terms of gender distribution, male athletes had a slightly higher injury rate (50.6%) than female athletes (45.2%).

Another concerning finding was the reliance on informal treatments or neglect of injuries. Athletes often resorted to self-medication or advice from peers and coaches rather than seeking professional medical care. This behavior delays recovery and heightens the risk of chronic complications. It is emphasized that in India, limited access to specialized sports medicine facilities and high treatment costs deter athletes from seeking timely intervention.

Psychological aspects also play a crucial role in injury recovery and recurrence. Competitive pressure often leads athletes to return to play prematurely, aggravating the injury. It was observed that psychological readiness and support systems significantly influence rehabilitation outcomes [10]. The present study did not formally assess psychological factors, indicating an avenue for future research. While the difference was not statistically significant, it reflects global patterns where males are generally more exposed to high-intensity play and contact scenarios [8]. Nevertheless, emerging trends suggest increasing vulnerability among female athletes, particularly in sports like athletics and cricket, due to anatomical differences, hormonal fluctuations, and lower muscle mass [11].

The data also suggests a systemic gap in injury surveillance and reporting. None of the participating institutions maintained formal injury logs, relying instead on anecdotal records. This lack of structured data hampers early identification of injury trends and obstructs implementation of targeted prevention strategies [9].

Addressing these gaps requires a multi-pronged approach. Sports academies must integrate sports physicians and injury prevention specialists into training routines. Coaches should undergo mandatory certification in sports injury first aid and prevention. Moreover, implementing regular athlete assessments and conditioning programs can reduce injury incidence. Educational campaigns targeting athletes and their families on injury signs, prevention, and treatment protocols are also essential.

Finally, regional authorities should consider establishing a centralized injury surveillance system, collecting data across sports academies, schools, and local tournaments. Such a database would enable real-time injury tracking and inform evidence-based policy decisions.

The findings from this study affirm that sports injuries represent a significant health burden among athletes in the Lucknow region, with a prevalence rate of 48.5%. This aligns with other North Indian studies reporting injury rates between 45% and 55% in similar age groups and sports disciplines [7]. Lower limb injuries were the most common (61.3%), echoing global trends that associate sports like football and wrestling with ankle sprains, ACL tears, and hamstring strains [4].

It is emphasized that structured warm-up protocols reduce injury incidence by improving flexibility and muscle preparedness. The absence of physiotherapists and trained support staff during practice sessions likely exacerbates these risks.

However, Indian studies have increasingly highlighted rising injury vulnerability in female athletes due to anatomical and hormonal differences affecting joint stability and muscle recovery [11]. Addressing these gender-specific needs through personalized training and injury management is essential.

Another concern was the common reliance on informal or peer-recommended treatment strategies. Many athletes reported self-medicating or seeking advice from teammates, often delaying professional medical care. This behavior increases the risk of chronic issues and long-term disability.

Additionally, the study identified a complete lack of injury surveillance systems across all five surveyed academies. No standardized injury logs or reporting mechanisms were in place. This hinders early detection of recurring injury patterns and impedes targeted interventions. The WHO advocates for centralized injury registries to inform data-driven policy changes [9]. Establishing such systems in Lucknow's academies could vastly improve injury tracking and prevention.

Environmental and equipment-related factors also contribute to injury risk. Several participants cited poor ground conditions, lack of appropriate footwear, and outdated training equipment. These issues mirror findings by Kumar et al. [2], who noted that nearly 30% of training injuries in Indian academies stem from environmental or infrastructural inadequacies.

Educational interventions offer another preventive avenue. Many athletes lacked basic knowledge about stretching, hydration, overtraining, and injury signs. Implementing injury prevention education modules as part of routine training can empower athletes to make safer choices [14]. Notably, injuries occurred more frequently during training sessions rather than competitions. This observation suggests inadequacies in training protocols, such as insufficient warm-up routines, inappropriate workload, and inadequate rest intervals [15].

Athletes in high‑impact team sports such as football and wrestling experience notably high rates of severe injuries-wrestling registers approximately 1.73 injuries per 1,000 athlete‑exposures, while football follows at about 0.97 per 1,000, with sprains and knee and ankle injuries being most frequent [16].

In contrast, in field events and track athletes (e.g., athletics), hamstring strains are particularly common-accounting for nearly 29 % of injuries among sprinters-with recurrence rates ranging from 14 % up to 63 % depending on severity and rehabilitation practices [17].

Essential implications of the study

This is among the first structured, multi-academy investigations in the Lucknow region to: (i) Quantify injury prevalence across multiple sports disciplines using validated tools; (ii) Identify high-risk sports and anatomical regions; (iii) Reveal systemic gaps in injury surveillance and athlete medical care; and (iv) Provide actionable recommendations tailored to regional needs.

Recommendations

It is recommended to (i) Embed sports physicians, sports medicine core team members, and injury prevention specialists within training environments, (ii) Mandate coach certification in injury first aid and prevention, (iii) Introduce athlete education programs on injury recognition, hydration, and safe training practices [12], (iv) Improve training infrastructure and ensure sport-specific protective equipment, and (v) Develop a centralized injury registry for continuous surveillance and policy support.

Future directions

Longitudinal studies incorporating both physical and psychological risk factors are warranted. Evaluating the impact of structured warm-up programs, biomechanical training, and surveillance systems could provide evidence for scalable preventive interventions.

Strengths

This study represents the first multi-academy, region-specific assessment of sports injuries in Lucknow, conducted using a stratified random sampling approach to ensure proportional representation across a range of sports disciplines. Such a sampling strategy enhances the representativeness of the findings and reduces the risk of over-representing or under-representing particular sports. The use of a validated questionnaire, corroborated by on-site clinical examinations and medical record reviews, further strengthens the study by enhancing the accuracy of injury diagnosis. In addition, the study not only documented injury patterns but also identified systemic gaps in injury care, thereby providing evidence that can be used to develop targeted, actionable recommendations for both prevention and management.

Limitations

Despite its strengths, this study has several limitations that should be acknowledged when interpreting the findings. The reliance on self-reported injury histories introduces the potential for recall bias, particularly for minor or older injuries that athletes may have forgotten or considered too insignificant to report. Furthermore, selection bias may have occurred because the study included only athletes enrolled in formal sports academies. As a result, the findings may not be fully generalizable to athletes training in informal settings or participating in recreational sports. Another potential source of bias is underreporting, as cultural norms and competitive pressures may have discouraged athletes from disclosing injuries, especially if they feared losing playing time or jeopardizing their chances for selection in competitions.

Measurement limitations

While clinical verification of reported injuries was attempted, it was not always possible to confirm all cases through imaging or advanced diagnostic tools. This limitation may have led to misclassification bias, particularly in differentiating between injuries with overlapping clinical presentations, such as sprains and strains. The absence of objective diagnostic confirmation for all cases means that some injuries may have been overestimated or underestimated in terms of severity and classification.

## Conclusions

In conclusion, the burden of sports injuries in Lucknow is substantial and multifactorial. Addressing it requires collaborative efforts from sports academies, healthcare providers, policymakers, and athletes themselves. Institutional reforms, increased investment in sports medicine, and evidence-based training policies will be crucial to safeguard athlete health and optimize their performance. The insights from this study offer a step toward developing a safer and more scientifically guided athletic ecosystem in the region.

Future initiatives should include region-wide workshops, continuous medical education for sports trainers, and a strong emphasis on injury prevention in sports curricula. Public-private partnerships could also be leveraged to fund Sports Medicine and Injury and rehabilitation centers within major sports hubs. Moreover, collaborations with national sports bodies and universities could pave the way for more rigorous longitudinal studies, ensuring that injury patterns are understood not just in isolation but within the broader context of athlete development over time. Together, these efforts will contribute to a more sustainable, health-oriented sports culture in Lucknow and beyond. Ultimately, safeguarding the well-being of athletes is not just a medical or institutional responsibility it is a shared societal commitment. By fostering a culture where health is valued as highly as performance, Lucknow can set a benchmark for other regions, proving that excellence in sports and athlete welfare can grow hand in hand.

## Appendices

Questionnaire

https://docs.google.com/document/d/1nBDNasA9gahhCx21yGXMKLE8iEWMeg67/edit?usp=sharing&ouid=105826173722118727044&rtpof=true&sd=true
